# Real-Time Monitoring for BDS Signal-In-Space Anomalies Using Ground Observation Data

**DOI:** 10.3390/s18061816

**Published:** 2018-06-04

**Authors:** Hu Jiang, Haitao Wang, Zemin Wang, Yunbin Yuan

**Affiliations:** 1 Chinese Antarctic Center of Surveying and Mapping, Wuhan University, Wuhan 430079, China; jianghu@whu.edu.cn (H.J.); zmwang@whu.edu.cn (Z.W.); 2 State Key Laboratory of Geodesy and Earth’s Dynamics, Institute of Geodesy and Geophysics, Chinese Academy of Sciences, Wuhan 430077, China; yybgps@whigg.ac.cn

**Keywords:** signal-in-space (SIS), User-Range Error (URE), BeiDou Navigation Satellite System (BDS), User Range Accuracy (URA), SIS anomalies

## Abstract

Signal-in-space (SIS) User Range Error (URE) is one of the major error sources for BeiDou Navigation Satellite System (BDS) applications and can reach tens of meters or even more. Therefore, real-time monitoring of SIS anomalies has a great realistic significance to guarantee the safety of users. According to an analysis of the BDS navigation messages, it showed that the User Range Accuracy (URA) index could not reflect the change of URE when it was abnormal. The conventional models using the relationship between URA and URE to monitor SIS anomalies are not suitable to the present BDS. Therefore, we use a prior information of SIS URE derived from ground observational data instead of URA to monitor BDS SIS anomalies. In order to realize the corresponding functions, we analysed the distribution of SIS UREs and obtained their prior models. Then, the monitoring threshold is determined using the prior models and a confidence interval instead of URA. The scheme was tested by applying to BDS SIS anomalies monitoring based on 13 ground tracking stations. The performance of this method was assessed by comparison with the satellite-health indicators from broadcast ephemeris. The results confirm that the method developed in this paper can rightly and timely detect abnormal SIS.

## 1. Introduction

The BeiDou Navigation Satellite System (BDS) was officially put into operation on 27 December 2012 and can overlap the Earth surface in 2020 s [1]. The service modes of BDS include the open service (OS) and the authorized service (AS) [2]. The Positioning, Navigation and Timing (PNT) performance of OS has basically reached or exceeded the design-performance requirements [3,4].

For most BDS OS users, real-time satellite orbits and clocks are derived from predicted ephemeris and clock parameters in navigation messages broadcast by BDS satellites. Signal-in-Space (SIS) errors arise primarily from imperfect estimation of a satellite orbit and clock error and are usually undetectable and uncorrectable for stand-alone OS users [5]. It is mainly described by the parameter of User Range Error (URE) that is the pseudorange inaccuracy attributable to the ground control and space vehicles and the one of major error sources affecting Global Navigation Satellite System (GNSS) applications [6]. The URE are dominated by the BDS Space and Ground Control Segment and do not include the error budget components assigned to the BDS User Segment such as the tropospheric or ionospheric modelling errors, multipath effects, and receiver noise. The constellation of BDS is different from other satellite navigation systems, including three types of orbits: medium earth orbits (MEO), inclined geostationary (IGSO), and geostationary orbits (GEO). SIS UREs of different type of BDS satellites are different [7,8]. According to the statistical analysis and evaluation on the broadcast ephemeris data, the SIS URE accuracy of the BDS is better than 2.5 m compared against the precise ephemeris data [9].

Nominally, OS users can assume that broadcast navigation message is reliable and the URE derived from a healthy SIS is at the meter level. In practice, however, SIS anomalies occurred occasionally and UREs of tens of meters or even more were observed, which could lead to hazardous misleading position solutions for unaugmented receivers [6]. Therefore, real-time monitoring of SIS anomalies has great importance for ensuring the safety of BDS users.

The study of monitoring SIS anomalies has long been conducted for Global Positioning System (GPS) [10,11,12,13,14], however, few studies have focused on BDS. Present detecting methods mainly depend on the relationship between User Range Accuracy (URA) in the broadcast navigation messages and URE to monitor SIS condition [6,13,14]. The URA is a conservative representation of the standard deviation of URE at the worst cast location on the earth [15]. In the case of GPS, the SPS SIS standard assures that the URE should be less than 4.42 times the upper bound on the URA value ($URA_{UB}$) for any healthy SIS during normal operation [16]. However, according to statistical analysis of BDS navigation messages for a long time, it showed that the BDS URA index values were constant, even in the case of satellite failure [17]. In the case of BDS, the specific definitions of the signal in SIS accuracy index parameters of broadcast navigation messages are not published in the current ICDs [18]. Therefore, the URA of BDS is unsuitable to construct a threshold to monitor the BDS SIS anomaly. Besides, the empirical values are also used as thresholds to detect BDS anomalies [19]. However, some error sources, such as multipath effect and receiver noise, are also closely related to monitoring stations, so the random model based on general hypothesis has defects that may result in false detection and false alarm in the practice use. In those cases, knowledge of characteristics of the BDS SIS URE has a great importance for monitoring the SIS anomalies.

The objective of the work presented here is to propose a scheme to monitor the BDS SIS anomalies using ground observation data. This program constructed a random model that was consistent with the actual URE based on the features of the BDS constellation and the observation conditions of the regional monitoring stations. According to the random models and a given confidence interval to obtain the threshold of URE instead of URA, the method is called BDS Ground SIS Monitoring (BDSGSISM). In this contribution, the BDS SIS UREs are derived from BDS satellites tracked by 13 ground stations of Multi-GNSS Experiment campaign (MGEX) of International GNSS Service (IGS) in the Asia-pacific region via deducting non-SIS errors from the total pseudorange errors in Day of Year (DOY) 36–56, 2017. In order to ensure the originality of broadcast navigation messages, the broadcast navigation messages are cleaned based on majority voting [13]. Then, we analyse SIS URE time series and calculate their distribution parameters. Based on the prior information of SIS URE, BDSGSISM is used to monitor the BDS healthy status for 27 days from 1 November 2015 to 27 November 2015 and presents the potential SIS anomalies during that time.

The remainder of this contribution is organized as follows: Section 2 introduces the methodology of BDSGSISM, the data source, the real-time broadcast ephemeris and the computation of SIS UREs. Section 3 analyses the characteristics of BDS SIS UREs. Section 4 presents two cases of SIS anomaly monitoring, and Section 5 provides concluding remarks.

## 2. Materials and Methods

### 2.1. BDS Ground SIS Monitoring Method

**Figure 1:**
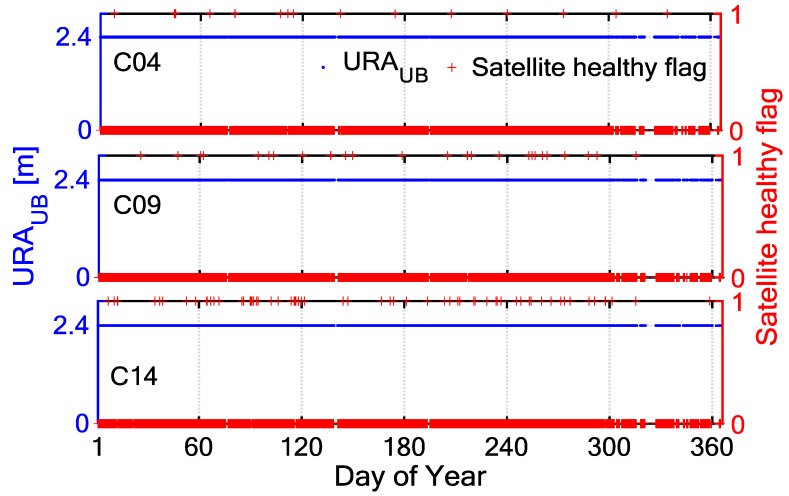
Time series of User Range Accuracy (URA) and satellite healthy flag (0: health, 1: anomaly) from BeiDou Navigation Satellite System (BDS) broadcast ephemeris for the C04 (**top**), C09 (**middle**), and C14 (**bottom**) during the year 2017.

Satellite-health status has specific markers in the broadcast ephemeris, however, if there is any sudden and unexpected failure, the Operational Control Segment (OCS) will guarantee a failure warning in the next broadcast ephemeris. This is insufficient for the users of BDS. Traditional SIS anomalies are monitored on the basis of the relationship between SIS UREs and URA as follows: if the SIS UREs are greater than $4.42\times URA_{UB}$, it can be determined that the satellite service is not guaranteed [12]. However, the URA index in BDS broadcast ephemeris is not equivalent to that of GPS [17]. Figure 1 shows the time series of $URA_{UB}$ and satellite healthy flag (0: health, 1: anomaly) from BDS broadcast ephemeris for the C04, C09 and C14 during the year 2017. The $URA_{UB}$ remains constant even if the satellites fail. In this case, a prior model of the SIS UREs of each satellite can be obtained through analysing the SIS UREs for a period of time from the selected tracking stations. Then, the thresholds of SIS UREs can be determined using the prior model and confidence level, instead of the URA. The specific processes are as follows:

1. Combining cleanly BDS ephemeris based on real-time raw data from tracking network.
2. The SIS UREs of all the satellites are calculated by the selected tracking stations’ observations.
3. The mean value and the standard deviation of the SIS UREs of each satellite observed by each station are calculated, as the prior information.
4. The threshold of the SIS UREs is calculated using Equation (1) based on a given confidence level and the prior information obtained in step 3.
  (1) $$Pr\left(\left|URE-\bar{URE}\right|\leq \kappa \left(Pr\right)\sigma_{URE}\right)=\alpha$$
  where $\bar{URE}$ is the mean value of the SIS UREs, $\sigma_{URE}$ is the standard deviation of the SIS UREs, and κ(Pr) is the quantile of the corresponding confidence α. In this step, we assume that the SIS UREs obey a Gaussian distribution.

In this paper, the monitored satellite-health status is represented by one of the following three numbers: −1 (abnormal), 0 (cannot be determined), and 1 (normal). Determining the health status of a satellite requires more than three tracking stations observing the satellites simultaneously; otherwise, the status of the satellite cannot be determined and is flagged 0. If more than three tracking stations detecting anomalies (URE exceeds a given confidence interval) at the same time then it is determined that the satellite cannot be used, and the status is flagged with −1. If a satellite is observed by more than three tracking stations at the same time and the SIS UREs for fewer than three tracking stations exceed the not-to-exceed (NTE) limit, then it is determined that the satellite is working normally, and the status is flagged 1.

The method may experience a missed alarm in the following situations: (1) when the satellite orbit is abnormal, but the projection of the orbital deviation is small in the monitoring service area, therefore, the monitoring stations cannot detect the abnormality. The effect of the anomaly is also not serious enough to cause dangerous misleading information for the users in the service area; (2) when a navigation satellite fails and misleads to users in the service area, but less than three monitoring stations simultaneously detect this anomaly in the service area. The probability of such event can be expressed as:

(2) $$P=P_{0}+P_{1}+P_{2}$$

where $P_{i}$ (i=0,1,2) is the probability of i stations that monitored the abnormal behavior of satellite when it suffered a failure. Then the probability of effectively monitoring the satellite failure is 1-P. The probability of a missed alarm is related to the tracking station density and the receiver stability. The denser the network of monitoring stations, the lower the probability of a missing alarm will be.

### 2.2. Data Source

**Figure 2:**
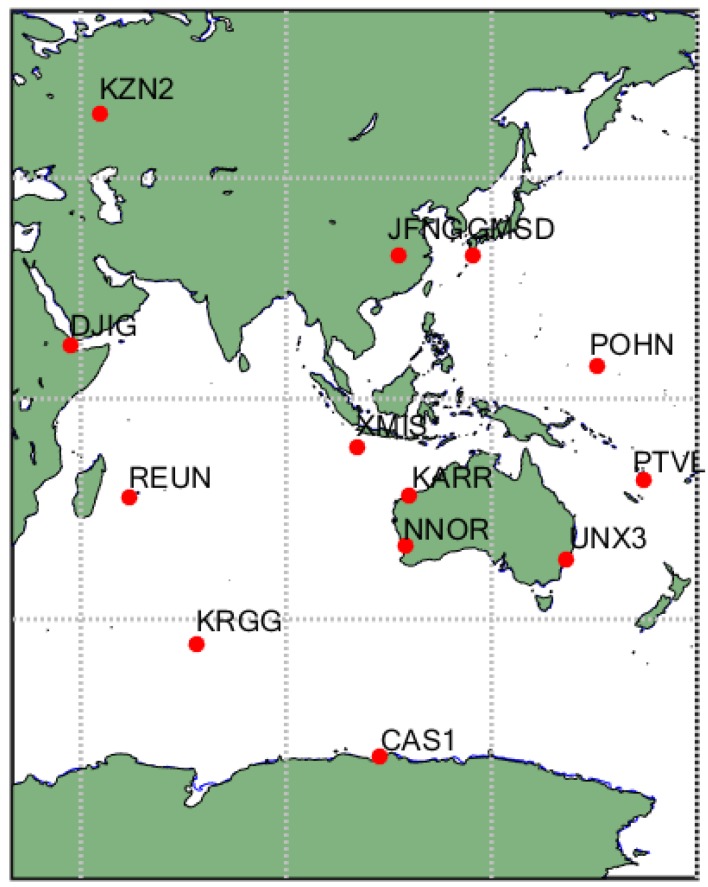
Multi-GNSS Experiment campaign (MGEX) tracking network used in this study.

In order to verify the reliability of the BDSGSISM, the BDS observation data from MGEX of International GNSS Service (IGS) for the years 2015 and 2017 have been used. As BDS mainly serves the Asia-pacific region, we selected 13 stations over the region and the nearby area as the experimental data sources. The specific information of these tracking stations including the tracking-station name, the receiver type, the antenna type and the BDS satellite is summarized in Table 1 and the distribution is shown in Figure 2.

The MGEX tracking station has the following advantages: (1) the selected tracking station can provide dual-frequency observation on B1 and B2 bands; (2) the MGEX tracking station can provide 1 Hz observation data, which is conducive to monitoring short-time satellite anomalies; and (3) we can obtain relatively long-time BDS observation data. To verify the reliability of the algorithm, we re-encoded broadcast ephemeris and observation data that have been broadcasted and transmitted in the form of real-time streams.

### 2.3. Combined Real-Time Broadcast Ephemeris

Due to accidental bad receiver data and various hardware/software bugs, a small proportion of the navigation data from the tracking stations have defects, such as losses, duplications, inconsistencies, discrepancies, and errors. Therefore, to accurately and effectively monitor BDS SIS anomalies, the navigation messages must be guaranteed authenticity. In this paper, we use a systematic methodology to combine BDS ephemeris using real-time data of simulation from multiple tracking stations.

Currently, the combined broadcast ephemeris provided by IGS and other institutions mostly take pseudorandom noise (PRN) and Time of Clock (TOC) (the meaning of these broadcast ephemeris parameters is same as in the reference [1], similarly hereinafter) as keywords to search and integrate ephemeris, but existing research [13] have shown that these two parameters have errors. In this paper, the broadcast ephemeris parameters are divided into robust parameters and fragile parameters [13]. The robust parameters are utilized to identify the equivalence of two navigation messages as follows: two navigation messages are deemed identical if and only if they agree on all the robust parameters, although their fragile parameters could be different. Most orbital and clock parameters in navigation messages are usually reported correctly, and even when errors occur, only a few stations agree on the same incorrect value [13]. These parameters are referred to as robust parameters, including: $C_{rs}$, Δn, $M_{0}$, $C_{uc}$, e, $C_{us}$, $\sqrt{A}$, $C_{ic}$, Ω, $C_{is}$, $i_{0}$, $C_{rc}$, ω, and $\dot{\Omega }$. On the contrary, other parameters, such as PRN, TOC, IDOE, and $\dot{i}$ are more likely to be erroneous, and when errors occur, several stations may make the same mistakes. These parameters are referred to as fragile parameters.

The broadcast ephemeris can be combined using the algorithm described below.

1. Broadcast ephemeris from a number of tracking stations are simultaneously received and added into the set O. Ensure that each satellite can be observed by a number of tracking stations (generally more than three tracking stations) at the same time.
2. For each navigation message e in O, if there is already a navigation message f in the set P (its database of different robust parameters) having the same robust parameters as e, then add the fragile parameters of e into f ’s database; otherwise, add e into P.
3. For each navigation message f in P, apply majority vote to each fragile parameter (except the Transmission Time of Message (TTOM)) according to f’s database, and record the number of stations that report f.

### 2.4. Computation of SIS UREs

The observation equation for the pseudorange observables can be modelled as:

(3) $$P_{r,i}^{s}=\rho_{r}^{s}+c\left(\delta t_{r}-\delta t^{s}\right)+T_{r}^{s}+\mu_{i}I_{r,1}^{s}+\varepsilon$$

where superscript *s*, subscripts *r* and i denote a specific satellite, receiver, and frequency band, respectively; $P_{r,i}^{s}$ is the raw code observation; $\rho_{r}^{s}$ is the geometric distances; $\delta t_{r}$ and $\delta t^{s}$ are the clock offsets for receiver and satellite, respectively; $T_{r}^{s}$ is the tropospheric delay; $I_{r,1}^{s}$ is the slant ionospheric delay on B1 signal and $\mu_{i}={\left(f_{1}/f_{i}\right)}^{2}$ is a coefficient related to the frequency, in which $f_{i}$ denotes the frequency of B_i_ signal; ε are the observation noise and unmodeled effects.

For the real-time users computing satellite orbits and clock offsets based on broadcast navigation messages, the geometric distance and satellite clock offsets can be written as:

(4) $$\{\begin{array}{c} \rho_{r}^{s}=\hat{\rho_{r}^{s}}+\varphi^{s}\\ \delta t^{s}=\hat{\delta t^{s}}+\phi^{s} \end{array}$$

where $\hat{\rho_{r}^{s}}$ is the geometric distance derived from broadcast ephemeris, $\varphi^{s}$ is the broadcast ephemeris error projected onto the line-of-sight (LOS) from a BDS satellite to a receiver, $\hat{\delta t^{s}}$ is the broadcast satellite clock offset and $\phi^{s}$ is the broadcast clock error.

Based on Equations (3) and (4), we can obtain the SIS URE, that is [6]

(5) $$SIS\;URE_{r,i}^{s}=c\phi^{s}-\varphi^{s}=\hat{\rho_{r}^{s}}+c\left(\delta t_{r}-\hat{\delta t^{s}}\right)+T_{r}^{s}+\mu_{i}I_{r,1}^{s}+\varepsilon -P_{r,i}^{s}.$$

In order to obtain the SIS URE, $T_{r}^{s}$ is derived from Saastamoinen model [20], $\hat{\rho_{r}^{s}}$ is computed from the broadcast ephemeris and the receiver position obtained from the “igs.snx” file (The file contains a summary of the station logs and is maintained at the IGS central bureau.) and $\hat{\delta t^{s}}$ is computed based on broadcast navigation messages. However, the slant ionospheric delay and receiver clock offset are estimated using least squares estimation. The detailed derivation is as follows:

(6) $${\left[\begin{array}{ccccc} c\delta t_{r} & I_{r,1}^{1} & I_{r,1}^{2} & \cdots & I_{r,1}^{n} \end{array}\right]}^{T}={\left(A^{T}WA\right)}^{-1}A^{T}WL$$

where $A=\left[\begin{array}{ccccc} 1 & -\mu_{1} & 0 & \cdots & 0\\ 1 & -\mu_{2} & 0 & \cdots & 0\\ 1 & 0 & -\mu_{1} & \cdots & 0\\ 1 & 0 & -\mu_{2} & \cdots & 0\\ \vdots & \vdots & \vdots & \vdots & \vdots \\ 1 & 0 & \cdots & 0 & -\mu_{1}\\ 1 & 0 & \cdots & 0 & -\mu_{2} \end{array}\right]$, $L=\left[\begin{array}{c} P_{r,1}^{1}-\hat{\rho_{r}^{1}}+c\hat{\delta t^{1}}-T_{r}^{1}\\ P_{r,2}^{1}-\hat{\rho_{r}^{1}}+c\hat{\delta t^{1}}-T_{r}^{1}\\ P_{r,1}^{2}-\hat{\rho_{r}^{2}}+c\hat{\delta t^{2}}-T_{r}^{2}\\ P_{r,1}^{2}-\hat{\rho_{r}^{2}}+c\hat{\delta t^{2}}-T_{r}^{2}\\ \vdots \\ P_{r,1}^{n}-\hat{\rho_{r}^{n}}+c\hat{\delta t^{n}}-T_{r}^{n}\\ P_{r,1}^{n}-\hat{\rho_{r}^{n}}+c\hat{\delta t^{n}}-T_{r}^{n} \end{array}\right]$, $W=diag(\left[\begin{array}{ccccccc} \sin^{2}(el_{r}^{1}) & \sin^{2}(el_{r}^{1}) & \sin^{2}(el_{r}^{2}) & \sin^{2}(el_{r}^{2}) & \cdots & \sin^{2}(el_{r}^{n}) & \sin^{2}(el_{r}^{n}) \end{array}\right])$, diag() is a function of diagonal matrices, and el is the satellite elevation angle.

## 3. BDS SIS URE Analysis and Discussion

For the BDS, and SIS URE the pseudorange inaccuracy, attributable to the ground control and the space vehicles, is one of the major error sources affecting BDS application. SIS URE refers to errors caused by satellite segment, including satellite ephemeris and clock errors, satellite antenna variations, and signal imperfections. However, SIS anomalies are mainly caused by satellite ephemeris and clock errors because antenna variations and signal imperfections are at a level of millimeter or centimeter [21]. As SIS anomalies occur occasionally and UREs of tens of metres or even more have been observed, knowledge of the SIS UREs is of great importance for developing GNSS SIS-monitoring systems.

**Figure 3:**
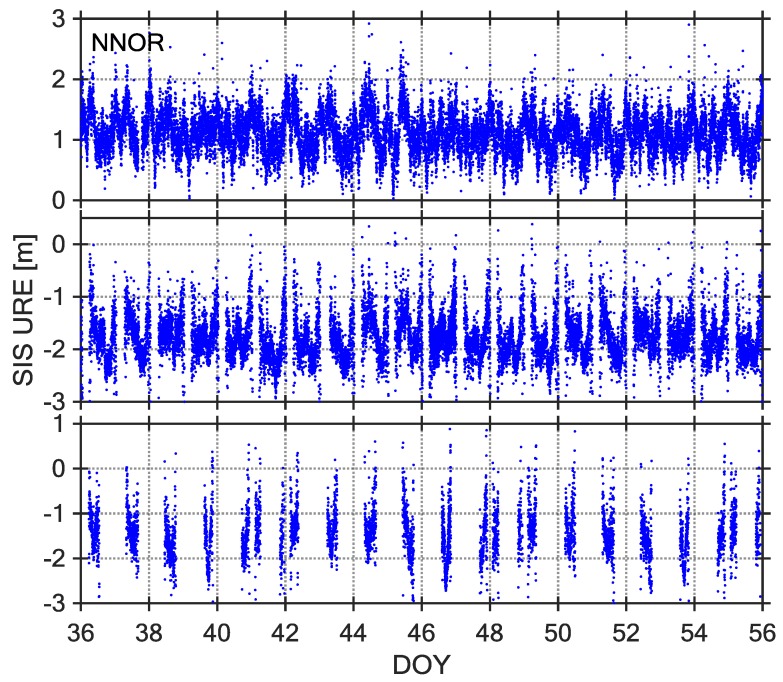
Signal-in-Space-User Range Error (SIS-URE) time series of Station NNOR, C04 (**top**), C09 (**middle**) and C14 (**bottom**), DOY 036–064, 2017.

**Figure 4:**
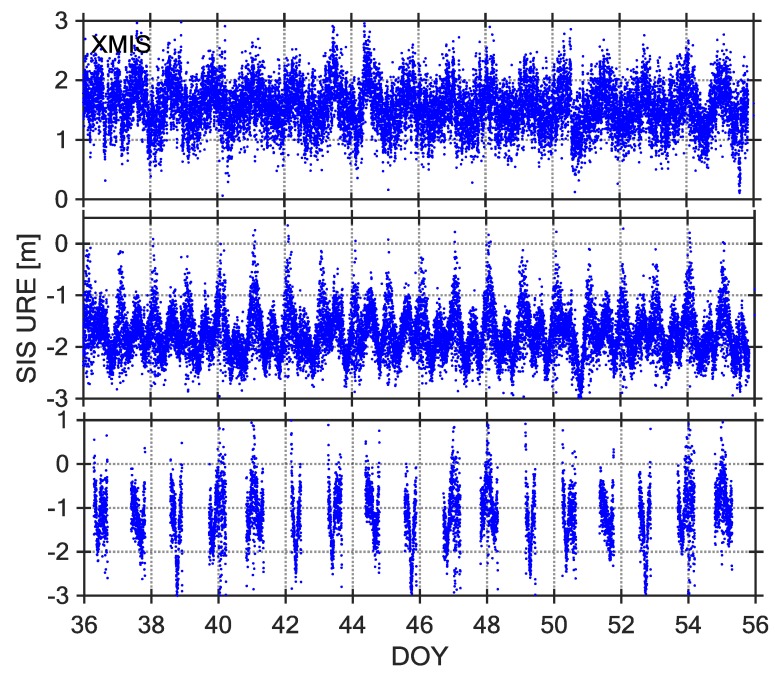
SIS-URE time series of Station XMIS, C04 (**top**), C09 (**middle**) and C14 (**bottom**), DOY 036–056, 2017.

**Figure 5:**
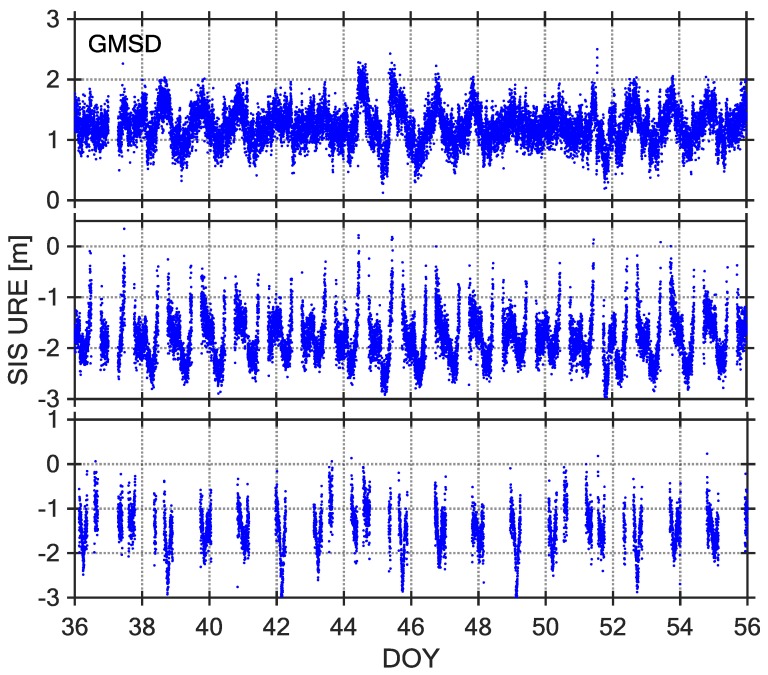
SIS-URE time series of Station GMSD, C04 (**top**), C09 (**middle**) and C14 (**bottom**), DOY 036–056, 2017.

The SIS UREs can be utilized to identify the satellite fault, and their description and assessment is very significant for BDS-SIS monitoring [7]. At present, most of the existing research assumes that the SIS UREs follow a Gaussian distribution with a mean value of 0 [22]. However, some researches have revealed that the code-peseudorange measurements of the MEO and IGSO satellites of the BDS exist nonzero mean values and have a certain relationship with the satellite elevation angle [23,24]. We have calculated the BDS SIS UREs based on the elevation weighting, as shown in Figure 3, Figure 4 and Figure 5.

Observations from 21-day intervals of DOY 036–056, 2017 were selected as the core data sets for studying BDS SIS UREs. The Figure 3, Figure 4 and Figure 5 show three types of URE time series (C04, C09, and C14) observed by three stations (NNOR, XMIS, and GMSD) with different latitudes. According to the time series of SIS UREs, which are very similar across all stations, there are obvious characteristics: the time series of C04 and C09 are dominated by some sinusoidal waves, however, the time series of C14 are not continuous because the satellite cannot be continuously observed by tracking stations. According to a comparison between broadcast ephemeris data and precise ephemeris product, the broadcast orbit errors exit significant periodic fluctuation in radial, along, and cross direction [7]. Therefore, the fluctuations of SIS UREs are mainly caused by the broadcast orbit errors.

Although the SIS UREs are generally assumed to be zeroes-mean, the reality may be different [23,25]. To monitor the SIS anomalies, it is necessary to eliminate the periodic system error in the SIS UREs, so that they satisfy a Gaussian distribution. In the section, we utilize the Fourier series to model the SIS UREs for each satellite observed by selected tracking stations. As to SIS UREs after eliminating the trend term, the traditional method can be used to monitor the health status of the satellite. Based on the long-term observation data, the period and amplitude of the UREs are analyzed, and the finite-term Fourier trigonometric-series model is established for the non-random term of the UREs. In this paper, we use the URE data during the recent 14 days to obtain the Fourier-series model and forecast the next seven days of the UREs. Assuming that the URE time series (y) can be expressed in the form of Equation (7) in the time domain, it can be represented by a set of independent forms, such as the Fourier trigonometric-series function, given in Equation (8).

(7) $$y^{T}=[\begin{array}{cccc} y_{1} & y_{2} & \cdots & y_{m} \end{array}]$$

(8) $$\{\begin{array}{c} E\{y(t)\}=A_{0}+\sum_{k=1}^{q}A_{k}x_{k}\\ A_{k}=\left[\begin{array}{cc} \cos\omega_{k}t_{1} & \sin\omega_{k}t_{1}\\ \cos\omega_{k}t_{2} & \sin\omega_{k}t_{2}\\ \vdots & \vdots \\ \cos\omega_{k}t_{m} & \sin\omega_{k}t_{m} \end{array}\right],x_{k}=\left[\begin{array}{c} a_{k}\\ b_{k} \end{array}\right] \end{array}.$$

where $A_{0}$ is the mean value of the coefficient and is a constant term; q is the number of trigonometric functions; $\omega_{k}$ is the frequency of trigonometric function k, corresponding to the period of time series $y^{T}$; and $a_{k},b_{k}$ are the model coefficients to be estimated. If $a_{k},b_{k}$ and $\omega_{k}$ are known, then y can be calculated at any time.

The prior models of SIS UREs can be written as:

(9) $$\{\begin{array}{c} \bar{URE^{\prime }}=\frac{1}{n}\sum_{i=1}^{n}(URE_{i}-URE_{i}^{f})\\ \sigma_{URE^{\prime }}=\sqrt{\frac{1}{n}\sum_{i=1}^{n}(URE_{i}-URE_{i}^{f}{)}^{2}} \end{array}$$

where $URE_{i}^{f}$ is computed from Fourier trigonometric-series function.

**Figure 6:**
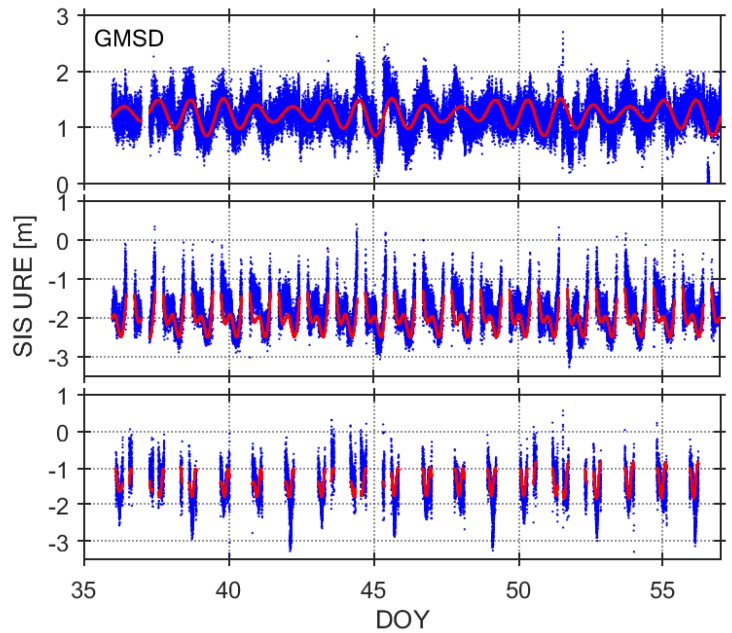
Examples of SIS-URE time series of Station GMSD, C04 (**top**), C09 (**middle**) and C14 (**bottom**), DOY 036–056, 2017, SIS UREs (**blue**) and Fourier models (**red**) on B1 band.

Representative results of such Fourier-series models are presented in Figure 6. The figure shows the results for three different types of satellites (PRN C04, C09, and C14) on B1 band, observed by GMSD station in 2017 (DOY 036–056). The red thin lines in Figure 6 represent the Fourier-series models for the SIS UREs (blue dots). The model parameters are determined according to the UREs during the 14 days (DOY 036–049), and used to forecast the next seven days (DOY 050–056).It can be seen that the Fourier-series models can fit the SIS UREs variation trend very well.

**Figure 7:**
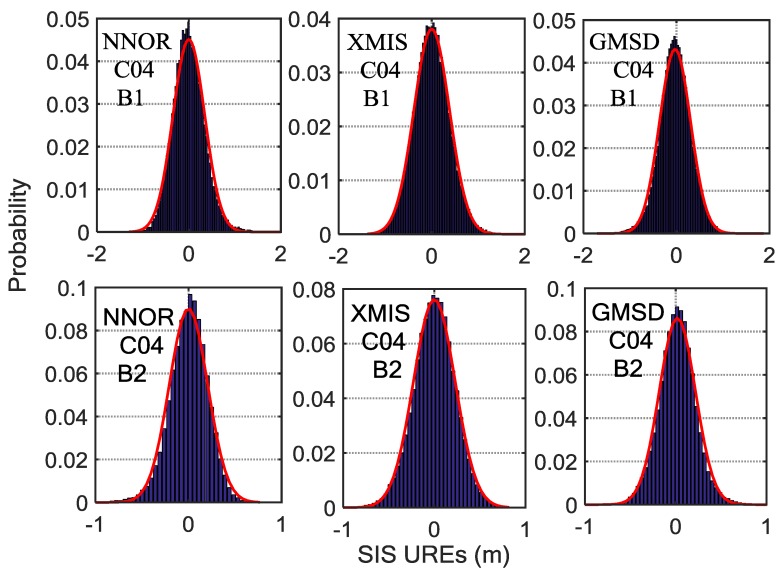
The histograms of C04 SIS UREs (m) after eliminating the trend term covering fourteen experimental days, overlaid with empirical normal probability-density function (PDF) curves (in red). Six subplots, arranged in two rows and three columns, show the results for three tracking stations (left column, NNOR; middle column, XMIS; right column, GMSD) and two frequencies (upper row, $B_{1}$ band; bottom row, $B_{2}$ band).

**Figure 8:**
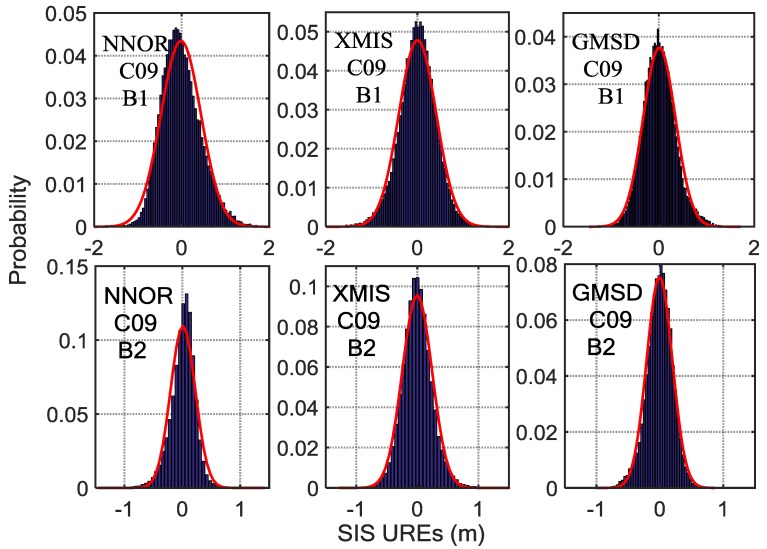
The histograms of C09 SIS UREs (m) after eliminating the trend term covering fourteen experimental days, overlaid with empirical normal probability-density function (PDF) curves (in red). Six subplots, arranged in two rows and three columns, show the results for three tracking stations (left column, NNOR; middle column, XMIS; right column, GMSD) and two frequencies (upper row, $B_{1}$ band; bottom row, $B_{2}$ band).

**Figure 9:**
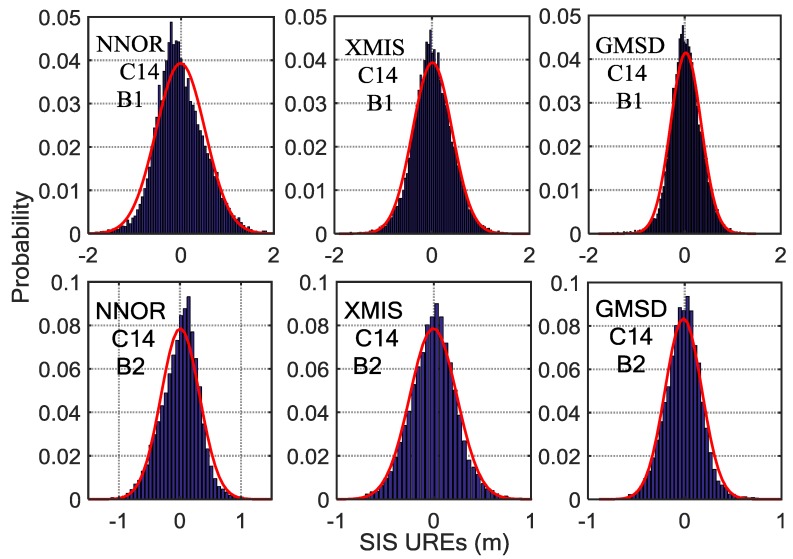
The histograms of C14 SIS UREs (m) after eliminating the trend term covering 14 experimental days, overlaid with empirical normal probability-density function (PDF) curves (in red). Six subplots, arranged in two rows and three columns, show the results for three tracking stations (left column, NNOR; middle column, XMIS; right column, GMSD) and two frequencies (upper row, $B_{1}$ band; bottom row, $B_{2}$ band).

By analyzing the SIS UREs (from which the periodic system error has been eliminated) from October 2016 to March 2017, there is a certain difference among the means and the standard deviations of the different satellites at different frequencies observed by different stations, as shown in Figure 7, Figure 8 and Figure 9. Besides, the statistical results of the SIS UREs (m) after eliminating the trend term are shown in Table 2. The overall level of the difference depends on certain characteristics of the signal itself and the tracking performance of the specific receiver and the antenna quality with respect to its multipath sensitivity [6]. Furthermore, the surroundings of the receiving antenna and its multipath contamination affect the SIS UREs values [26,27]. The computation method of SIS UREs also results in the differences at different frequencies. In addition, the observation accuracy of different BDS satellite is also different, varying from 0.5 m to 1.8 m [7]. Therefore, various models instead of the same model are preferable to monitor SIS UREs for different stations, satellites and frequencies.

## 4. Case Studies of BDS SIS Anomalies

SIS anomaly monitoring requires real-time data streams of the tracking stations, however, for various reasons, the current MGEX tracking stations have not provided the BDS real-time data streams. In order to verify the reliability of the proposed algorithm, we re-encoded the broadcast ephemeris and the observation data that have been broadcasted and transmitted in the form of real-time streams. Through processing the simulated real-time data streams, some potential SIS anomalies are found and listed in Table 3. The parameter κ(Pr) in Equation (1) is set to 4.42 based on the relationship between URA and URE as well as integrity monitoring requirements. An SIS anomaly is claimed when both of the following conditions are fulfilled: (1) a satellite is observed by more than three tracking stations and its SIS UREs derived from more than three stations exceed the given confidence interval at the same time; (2) the unhealthy flag of the broadcast ephemeris is not set on.

**Figure 10:**
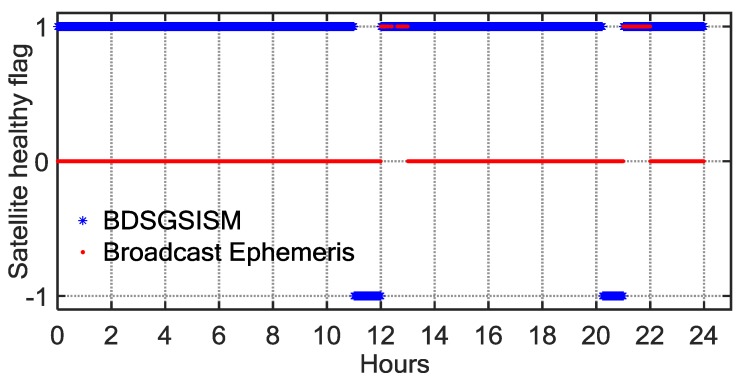
Satellite-health flag as a function of BDT, determined using two methods (blue *, BDS Ground SIS Monitoring (BDSGSISM); red dots, broadcast ephemeris) on day 346 of 2015 for BDS satellite C02.

For the monitoring results in Table 3, in-depth case studies of the C02 anomaly on 12 December 2015 are presented. Figure 10 shows the health statuses of C02, monitored by BDSGSISM (blue asterisks), compared with the health statuses given by the broadcast ephemeris (red dots). As illustrated in Figure 10, the C02 satellite occurred two periods of abnormal performances on that day based on the information provided by the BDSGSISM as well as the broadcast ephemeris. Although the two results showed that each anomaly lasted for one hour, starting times of each anomaly are not consistent for two methods. This example indicates that at least one monitoring result is wrong. Therefore, we analysed the SIS URE time series of C02 and found that C02 could have been simultaneously observed by six to seven tracking stations at the two periods of anomaly, and which all monitored these two anomalies. The monitoring results of BDSGSISM are better than those from the broadcast ephemeris since it timely and accurately detected the anomaly.

**Figure 11:**
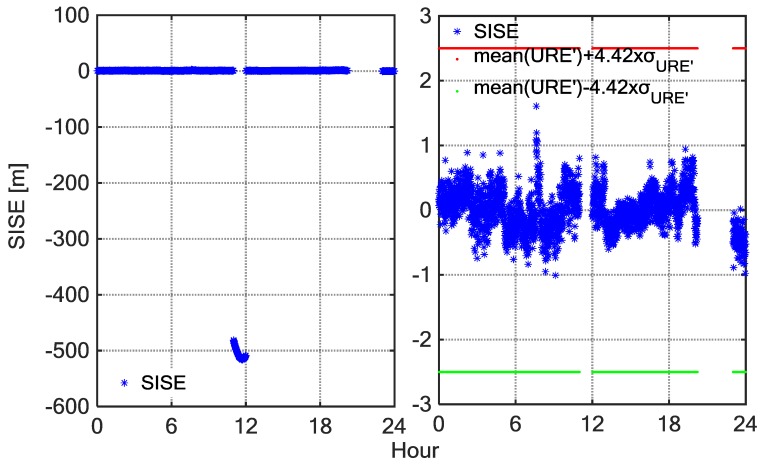
SIS-URE after eliminating the trend term time series as a function of BDT on day 346 of 2015 for BDS satellite C02 tracked by NNOR. Subplot (**left**) corresponds to the tracking data of satellite C02 by NNOR for the whole day, and subplot (**right**) corresponds to the tracking data of satellite C02 by NNOR with the anomalous periods removed.

Figure 11 shows the SIS UREs time series of C02 observed by a MGEX station, NNOR on DOY 346, 2015. The left panel shows the SIS URE time series of C02 whole day tracked by NNOR, and the right panel shows the SIS URE of C02 by NNOR with the anomalous periods removed. The first anomaly experienced by NNOR started at 11:00 and ended at 12:00. The UREs suddenly increased and exceeded the confidence interval during this period. The anomaly may have resulted from the unplanned operations. Therefore, broadcast ephemeris cannot alarm on time. When anomalies were detected, the operation control centre immediately adjusted the health status of the satellite, but the TTA (Time to Alarm) of the broadcast ephemeris was too long, which may impact some applications requiring the safety. The BDSGSISM can make up for this shortcoming.

**Figure 12:**
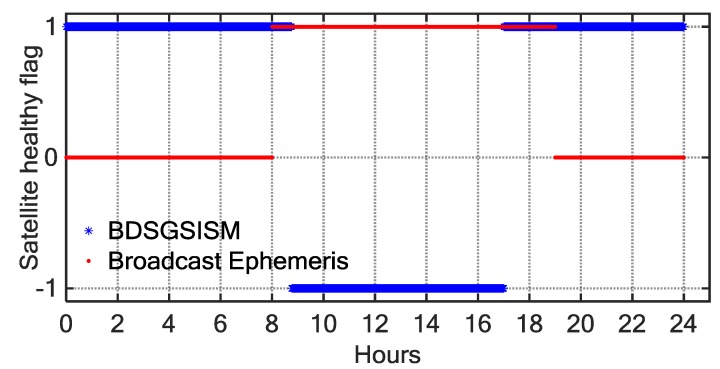
Satellite-health flag as a function of BDT, determined using two methods (blue *, BDSGSISM; red dots, broadcast ephemeris) on day 359 of 2015 for BDS satellite C10.

**Figure 13:**
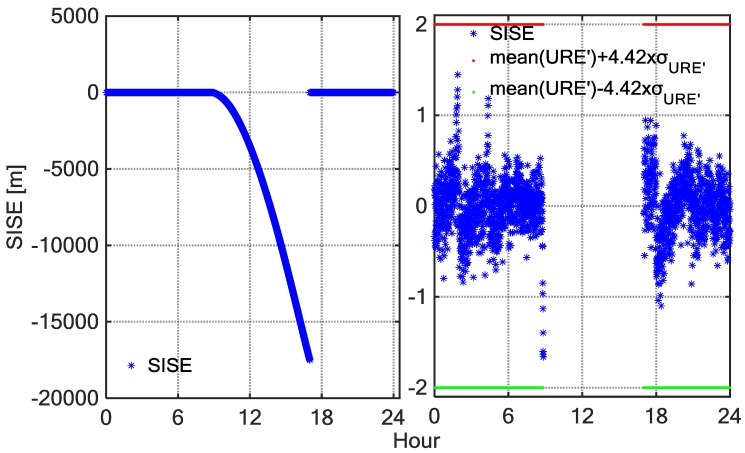
SIS-URE after eliminating the trend term time series as a function of BDT on day 359 of 2015 for BDS satellite C10 tracked by XMIS. Subplot (**left**) corresponds to the tracking data of satellite C10 by XMIS for the whole day, and subplot (**right**) corresponds to the tracking data of satellite C10 by XMIS with the anomalous periods removed.

According to the analysis of the monitoring results, it can be found that in the BDS broadcast ephemeris there exist missed detection as well as false alarms. Figure 12 shows the health statuses of C10 monitored by BDSGSISM (blue asterisks) compared with the health statuses provided by the broadcast ephemeris (red dots) in 25 December 2015 (DOY 359, 2015). We can find that the health statuses monitored by BDSGSISM do not match well with those provided by the broadcast ephemeris. According to the broadcast ephemeris, the anomaly started at 08:00 and ended at 19:00, indicating that the anomaly lasted for 11 h. However, the monitoring result of BDSGSISM shows that the anomaly started at 08:46 and ended at 17:00, indicating that the anomaly lasted for only 8.24 h. Therefore, we analysed the SIS UREs observed by the tracking-station network. Between 08:00 and 08:46, there were 9 to 11 tracking stations to observe C10 and all show it to be operating normally. At about 08:46, the UREs began to increase with time, and exceeded confidence intervals, as three stations simultaneously monitored the anomaly, and this lasted for only 8.24 h, four to eight stations simultaneously monitored the anomaly during the period. At about 17:00, the satellite restored healthy. As illustrated in Figure 13, we can find that the SIS UREs exceeded the confidence interval at 08:46 and gradually increased until returning to normal at 17:00.

## 5. Conclusions

BDS SIS anomaly monitoring is essential to guarantee the safety of the user’s life and property. SIS anomaly is one of the major risk sources for user’s PNT services. However, the traditional monitoring method of SIS anomaly cannot be applied to BDS due to invalid URA parameter in BDS broadcast ephemeris. Therefore, it is essential to take an effective approach to monitor the SIS anomaly for the wide application of BDS. In this paper, we propose BDSGSISM method based on the prior information (mean and variance) of SIS UREs to monitor BDS SIS anomalies.

In order to guarantee authenticity of broadcast ephemeris, the cleaned navigation messages are combined based on broadcast ephemeris received from a number of tracking stations. According to analysing BDS SIS UREs, it shows that the SIS UREs do not obey a Gaussian distribution due to systemic errors. Therefore, we utilize Fourier series to model the SIS UREs to eliminate the periodic system error, making them obey a Gaussian distribution and satisfy the precondition of SIS-anomaly monitoring. Besides, we calculate the SIS URE’s means and variances for all satellites observed by selected tracking stations and find that there are differences in these values for different satellites. Finally, the NTE threshold is determined by the prior information of SIS UREs and a given confidence interval, instead of URA.

To verify the reliability of BDSGSISM, the collected BDS data of 57 days (DOY 305–361, 2015) were analysed using BDSGSISM and presented the potential signal-in-space anomalies. According to a comparison between the satellite health states obtained by BDSGSISM monitoring and those given by broadcast ephemeris, it can be found that there exist false alarms and missed detections in the results obtained by broadcast ephemeris, however, BDSGSISM can quickly and accurately find the abnormal satellite and overcome the shortcoming.

## Figures and Tables

**Table 1:** MGEX tracking-station information in this study.

Tracking Stations	Receiver Type	Antenna Type	PRN of Tracking
CAS1	Trimble NETR9	LEIAR25.R3	C01 C03 C06 C07 C08 C09 C10 C11 C12 C14
DJIG	Trimble NETR9	TRM59800.00	C02 C03 C05 C06 C07 C08 C09 C10 C11 C12 C14
GMSD	Trimble NETR9	TRM59800.00	C01 C02 C03 C04 C06 C07 C08 C09 C10 C11 C12 C14
KARR	Trimble NETR9	TRM59800.00	C01 C03 C04 C05 C06 C07 C08 C09 C10 C11 C12 C14
KRGG	Leica GR10	LEIAR25.R4	C02 C03 C05 C06 C07 C08 C09 C10 C11 C12 C14
KZN2	Trimble NETR9	TRM59800.00	C02 C05 C06 C07 C08 C09 C10 C11 C12 C14
JFNG	Trimble NETR9	TRM59800.00	C01 C02 C03 C04 C05 C06 C07 C08 C09 C10 C11 C12 C14
NNOR	SEPT POLARX4	SEPCHOKE_MC	C01 C02 C03 C04 C05 C06 C07 C08 C09 C10 C11 C12 C14
POHN	Trimble NETR9	TRM59800.00	C01 C03 C04 C06 C07 C08 C09 C10 C11 C12 C14
PTVL	Trimble NETR	TRM59800.00	C01 C03 C04 C06 C07 C08 C09 C10 C11 C12 C14
REUN	Trimble NETR9	TRM55971.00	C02 C03 C05 C06 C07 C08 C09 C10 C11 C12 C14
UNX3	SEPT ASTERX3	LEIAR25.R3	C01 C03 C04 C06 C07 C08 C09 C10 C11 C12 C14
XMIS	Trimble NETR9	TRM59800.00	C01 C03 C04 C05 C06 C07 C08 C09 C10 C11 C12 C14

**Table 2:** Statistical results of the SIS UREs (m) after eliminating the trend term covering 14 experimental days.

Station	Frequency	C04		C09		C14	
		Mean/m	STD/m	Mean/m	STD/m	Mean/m	STD/m
NNOR	B1	0	0.35	−0.02	0.46	−0.01	0.53
	B2	0	0.21	0.01	0.21	0.01	0.32
XMIS	B1	0	0.38	0	0.41	0.01	0.40
	B2	0	0.23	0	0.24	0	0.24
GMSD	B1	0.05	0.33	0	0.37	0.02	0.32
	B2	0.06	0.20	0	0.21	−0.01	0.19

**Table 3:** List of Potential SIS Anomalies from 1 November 2015 to 27 December 2015.

Satellite	PRN	Date	Start Time	Duration (Minutes)
G04	C04	6 November 2015	07:29	32
I01	C06	7 November 2015	03:23	41
I02	C07	7 November 2015	11:17	50
M03	C11	8 November 2015	15:15	44
G06	C02	11 November 2015	04:08	51
G03	C03	11 November 2015	07:40	21
G04	C04	14 November 2015	02:29	93
I05	C10	14 November 2015	12:00	20
I02	C07	14 November 2015	15:30	47
M03	C11	14 November 2015	17:55	365
M03	C11	15 November 2015	00:01	180
G06	C02	15 November 2015	05:18	42
G06	C02	19 November 2015	06:10	51
G06	C02	21 November 2015	07:07	53
G06	C02	27 November 2015	01:44	29
G06	C02	1 December 2015	00:25	81
I01	C06	2 December 2015	01:42	112
G06	C02	7 December 2015	20:08	52
G03	C03	8 December 2015	07:59	61
G04	C04	8 December 2015	21:23	38
G06	C02	9 December 2015	19:57	63
I01	C06	11 December 2015	06:40	62
I02	C07	11 December 2015	09:02	84
G06	C02	12 December 2015	11:00	120
G06	C02	13 December 2015	01:50	28
G04	C04	14 December 2015	08:27	32
G06	C02	15 December 2015	07:12	48
M04	C12	15 December 2015	07:38	25
G06	C02	16 December 2015	06:33	31
G05	C05	18 December 2015	04:15	43
G06	C02	20 December 2015	10:53	29
I01	C06	23 December 2015	04:44	32
M04	C12	23 December 2015	07:10	23
I02	C07	25 December 2015	12:45	29
G06	C02	27 December 2015	05:23	25
